# Assessment of plasmids for relating the 2020 *Salmonella enterica* serovar Newport onion outbreak to farms implicated by the outbreak investigation

**DOI:** 10.1186/s12864-023-09245-0

**Published:** 2023-04-04

**Authors:** Seth Commichaux, Hugh Rand, Kiran Javkar, Erin K. Molloy, James B. Pettengill, Arthur Pightling, Maria Hoffmann, Mihai Pop, Victor Jayeola, Steven Foley, Yan Luo

**Affiliations:** 1 Center for Food Safety and Nutrition, Food and Drug Administration, Laurel, MD USA; 2 Center for Food Safety and Nutrition, Food and Drug Administration, College Park, MD USA; 3 Center for Bioinformatics and Computational Biology, University of Maryland, College Park, MD USA; 4 Biological Science Graduate Program, University of Maryland, College Park, MD USA; 5 Department of Computer Science, University of Maryland, College Park, MD USA; 6 Joint Institute for Food Safety and Applied Nutrition, University of Maryland, College Park, MD USA; 7 Food and Drug Administration, National Center for Toxicological Research, Jefferson, AR USA

**Keywords:** Source tracking, Molecular epidemiology, *Salmonella enterica*, *Salmonella enterica* Newport, Pangenome, Mobilome, Plasmid

## Abstract

**Background:**

The *Salmonella enterica* serovar Newport red onion outbreak of 2020 was the largest foodborne outbreak of *Salmonella* in over a decade. The epidemiological investigation suggested two farms as the likely source of contamination. However, single nucleotide polymorphism (SNP) analysis of the whole genome sequencing data showed that none of the *Salmonella* isolates collected from the farm regions were linked to the clinical isolates—preventing the use of phylogenetics in source identification. Here, we explored an alternative method for analyzing the whole genome sequencing data driven by the hypothesis that if the outbreak strain had come from the farm regions, then the clinical isolates would disproportionately contain plasmids found in isolates from the farm regions due to horizontal transfer.

**Results:**

SNP analysis confirmed that the clinical isolates formed a single, nearly-clonal clade with evidence for ancestry in California going back a decade. The clinical clade had a large core genome (4,399 genes) and a large and sparsely distributed accessory genome (2,577 genes, at least 64% on plasmids). At least 20 plasmid types occurred in the clinical clade, more than were found in the literature for *Salmonella* Newport. A small number of plasmids, 14 from 13 clinical isolates and 17 from 8 farm isolates, were found to be highly similar (> 95% identical)—indicating they might be related by horizontal transfer. Phylogenetic analysis was unable to determine the geographic origin, isolation source, or time of transfer of the plasmids, likely due to their promiscuous and transient nature. However, our resampling analysis suggested that observing a similar number and combination of highly similar plasmids in random samples of environmental *Salmonella enterica* within the NCBI Pathogen Detection database was unlikely, supporting a connection between the outbreak strain and the farms implicated by the epidemiological investigation.

**Conclusion:**

Horizontally transferred plasmids provided evidence for a connection between clinical isolates and the farms implicated as the source of the outbreak. Our case study suggests that such analyses might add a new dimension to source tracking investigations, but highlights the need for detailed and accurate metadata, more extensive environmental sampling, and a better understanding of plasmid molecular evolution.

**Supplementary Information:**

The online version contains supplementary material available at 10.1186/s12864-023-09245-0.

## Introduction

In 2020, the United States Food and Drug Administration (FDA) and the Centers for Disease Control and Prevention (CDC) responded to the largest *Salmonella* outbreak in over a decade. The outbreak was caused by a strain of *Salmonella enterica subspecies enterica* serovar Newport (from lineage III), referred to here as *Salmonella* Newport. *Salmonella* Newport is one of the top five serovars contributing to the approximately 80.3 million foodborne cases of Salmonellosis each year [1]. *Salmonella* Newport is composed of three polyphyletic lineages and is frequently associated with cattle [2]; however, it can colonize a wide range of animal (wild and domesticated) and plant species, providing it multiple reservoirs and multiple transmission routes to humans [3]. *Salmonella* Newport can also persist in the environment (e.g., in manure) for months [4].

The first cases from the outbreak were reported in June of 2020 and the CDC declared the end of the outbreak in October 2020, after causing nearly 2,000 illnesses in the United States and Canada. The investigation traced the outbreak from the food history of the sick patients to red onions grown on two farms in Holtville and Bakersfield California [5]. This *Salmonella* outbreak was soon followed in 2021 by another large outbreak (over 1,000 reported cases) of *Salmonella enterica* serovar Oranienburg that was linked to bulb onions imported from Mexico, lending urgency to understanding the chain of events that led to the outbreaks [6].

The leading hypothesis of the FDA from the on-site investigation of the onion farms was that contaminated irrigation water was used to grow the onions at the Holtville California farm [7]. Plausible sources of contamination were identified, such as sheep grazing on adjacent land and signs of animal intrusion (e.g., scat and large flocks of birds). After harvesting, the outbreak strain could have been transmitted to Bakersfield because many of the Holtville onions had been shipped to Bakersfield for packaging and distribution. Although the investigation occurred after the onions were packaged and distributed, visual observations of the packing house confirmed numerous opportunities for contamination, including signs of animal and pest intrusion, as well as food contact surfaces which had not been properly inspected, maintained, or cleaned [7].

In molecular epidemiology, whole genome sequencing data ( WGS) is a powerful tool for outbreak investigations and isolates presenting little genetic distance are likely linked in the transmission chain [8]. Single nucleotide polymorphism (SNP) analysis of the WGS data with the Center for Food Safety and Nutrition (CFSAN) SNP pipeline [9] determined that the clinical isolates were linked and formed a single, nearly-clonal clade (further referred to as the clinical clade), consistent with a single point source of contamination. SNP analysis also determined that none of the environmental *Salmonella enterica*, from 30 serovars, collected near and on the farms (further referred to as the farm isolates), matched the outbreak strain—preventing the conclusive identification of the outbreak source with the WGS data.

Here, we explored an alternative method for analyzing the WGS data driven by the hypothesis that isolates that had recently coexisted in the same microbiome (i.e., the outbreak strain and environmental isolates) might share plasmids related by recent horizontal transfer. Horizontal transfer is the movement of genetic material between the genomes of organisms, turning the tree of life into an evolutionary network [10, 11]. A common bioinformatic approach for detecting horizontal transfer events is to identify incongruences between the phylogeny of the lineages being analyzed and the phylogenies of the sequences suspected to be horizontally transferred [12]. Horizontal transfer is a fundamental source of genetic adaptation (such as antimicrobial resistance genes and metabolic pathways) to new environments and conditions for bacteria [10, 11]. Plasmids are small (generally between 1 and 200 kbp) intracellular genetic elements that can semi-independently replicate and are thought to be the most impactful source of rapid horizontal transfer in microbial communities [13, 14]. Multiple plasmids, representing various incompatibility types (Inc), have been observed in *Salmonella* Newport (i.e., IncA/C, IncR, IncI1, IncN, IncH, IncF, ColE1, IncP); however, plasmids are likely under-identified given that most were found while looking for genes conferring antimicrobial resistance [15–21].

As part of our study, we analyzed the pangenome—the totality of gene families found in a set of isolates—of the clinical isolates [22]. The pangenome can be subdivided into the core genes, which are common to all the genomes and the accessory genes which are only found in a subset. Further, pangenomes can be described as closed if the number of observed gene families approaches a limit as more genomes are sampled. In contrast, in an open pangenome, the number of genes continues to grow as more genomes are sampled. A recent study of the three polyphyletic lineages of *Salmonella* Newport found that each lineage had a closed pangenome with a large number of core genes (ranging from 3,489 to 3,820), indicating the lineages underwent limited horizontal transfer [23].

We hypothesized that if the reservoir population of the clinical clade existed in the Holtville and Bakersfield farm regions, there was the possibility that it interacted with the local microbial communities via horizontally transferred plasmids. Through the analysis of the 2020 *Salmonella* Newport onion outbreak, we first explored whether a highly related lineage of *Salmonella* Newport had a substantial accessory genome and plasmid diversity. Secondly, we sought to detect horizontally transferred plasmids that provided evidence the clinical and farm isolates had coexisted in the same microbiota.

## Results

### Whole genome SNP analysis of the clinical isolates

**Fig. 1:**
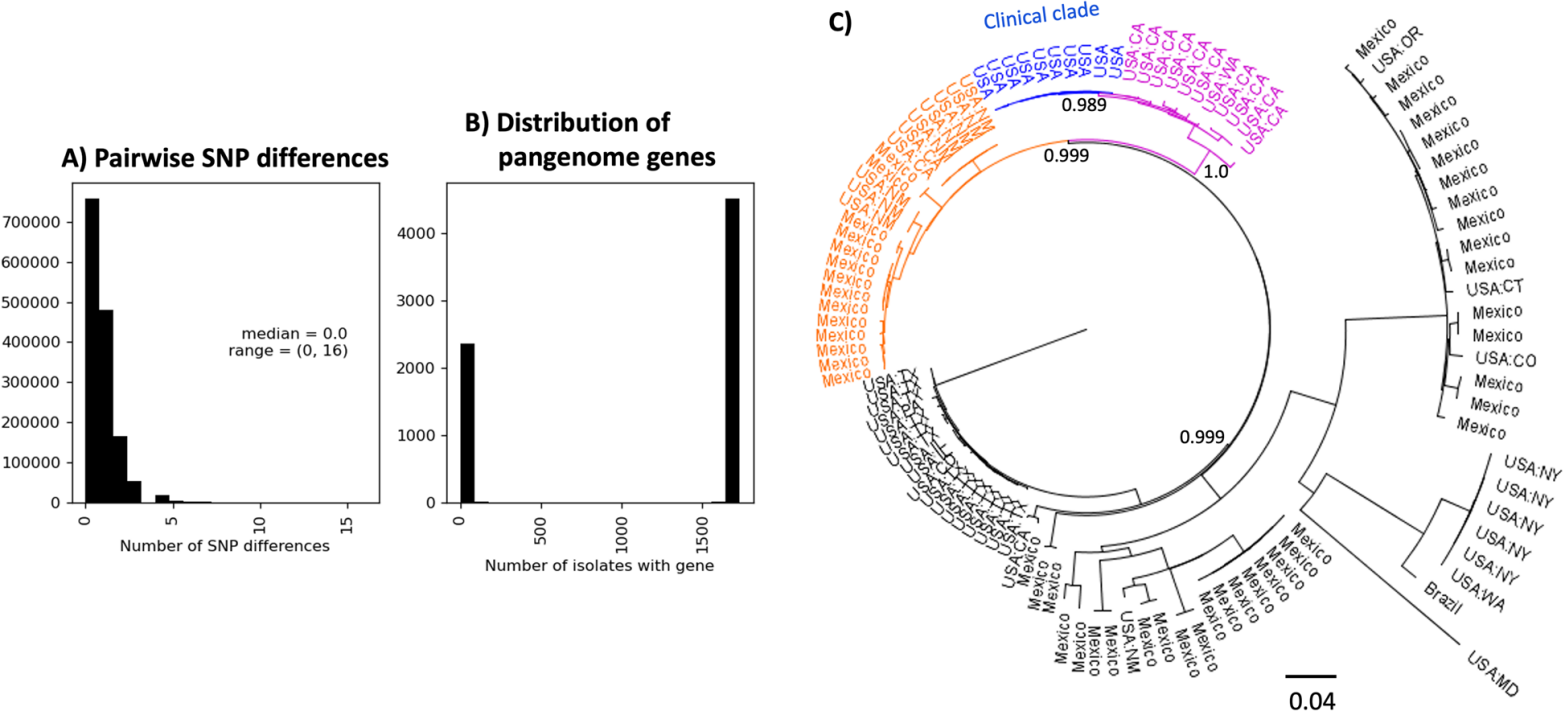
Characteristics of the 1,728 clinical isolates. **A** A histogram showing the number of pairwise SNP differences. **B** A histogram showing the number of isolates that carry each of the 6,976 pangenome genes. **C** Geography of the phylogenetic neighborhood of the clinical clade. The SNP-based maximum likelihood phylogeny contains all environmental isolates identified in the NCBI Pathogen Detection database within 1000 cgMLST alleles of the clinical clade. Only select bootstrap values that highlight the separation between the clinical and environmental isolates are shown for clarity. The clinical clade (blue), which is only represented by ten isolates here, is nested within a larger clade (magenta) of ten isolates collected from California and one isolate from Washington, collected between 2010 and 2017. The sister clade (orange) contains isolates from California, New Mexico, and Mexico collected between 2014 and 2020. The phylogeny was inferred using GARLI (with 1,000 bootstraps) with the SNP matrix generated by the CFSAN SNP Pipeline

Whole genome, reference-based Single nucleotide polymorphism (SNP) analysis confirmed the previous findings of the FDA and CDC outbreak investigation—the clinical clade formed a single, nearly-clonal clade. The pairwise number of SNP differences ranged from 0 to 16 with a median of 0 (Fig. 1A). For reference, the median assembly length for the clinical isolates was 4.8 Mbp. The phylogeny constructed from the SNP results revealed a single large polytomy which precluded any observations about the clustering of the clinical isolates by collection date or geographic location.

To assess if the clinical clade was associated with a geographical location, the previous SNP analysis was extended to include all closely related (within 1,000 cgMLST alleles of the clinical isolates) environmental isolates from the National Center for Biotechnology Information (NCBI) Pathogen Detection database (Fig. 1C). The most closely related isolates were mainly from California, New Mexico, and Mexico. The clinical isolates formed a subclade within the SNP phylogeny with 10 environmental isolates from California (seven isolated from almonds, one from parsley, two from environmental swabs) and one from Washington state (isolated from pistachios likely grown in California [24]). These isolates had been collected between 2010 and 2017 and were between 8 and 39 SNPs distant from the clinical isolates. Sister to this subclade were environmental isolates from California (comminuted beef), New Mexico (environmental swabs from an unknown source), and Mexico (mostly water samples from rivers, canals, and a reservoir, with one from a chicken caecum) collected between 2014 and 2020.

### The pangenome of the clinical clade had a substantial accessory genome

The core genome of the clinical clade was highly conserved, consistent with the low diversity observed in the SNP analysis. There were 4,399 core genes (genes occurring in 99% of the clinical isolates) which was near the median number of genes per genome (4,512 genes). The large and conserved core genome was accompanied by a substantial accessory genome containing 2,577 genes. These accessory genes were sparsely distributed, mostly occurring in 6% or less of the isolates, indicative of horizontal transfer (Fig. 1B).

### Plasmids of the clinical and farm isolates

It was found that ≥ 64% of the accessory genes were carried on plasmids. For the clinical isolates, 1,814 putative plasmid contigs were identified, belonging to 20 known plasmid types (Table 1). We also identified 373 putative plasmid contigs of unknown type. The typeable plasmids accounted for 1,590 genes of the clinical clade pangenome (16 core and 1,574 accessory genes) and ranged in size from approximately 1.6 kbp to 85 kbp long. The most observed plasmid type was the IncFII(S), occurring in all clinical isolates. Other plasmid types occurred infrequently. For example, the second most abundant plasmid type, IncI1-I(Gamma), was only identified in 27 (1.6%) clinical isolates. No clustering of the plasmids was observed in the whole genome SNP phylogeny due to it being a large, unresolved polytomy. Additionally, no plasmid was found to be significantly associated with hospitalization.

**Table 1:**
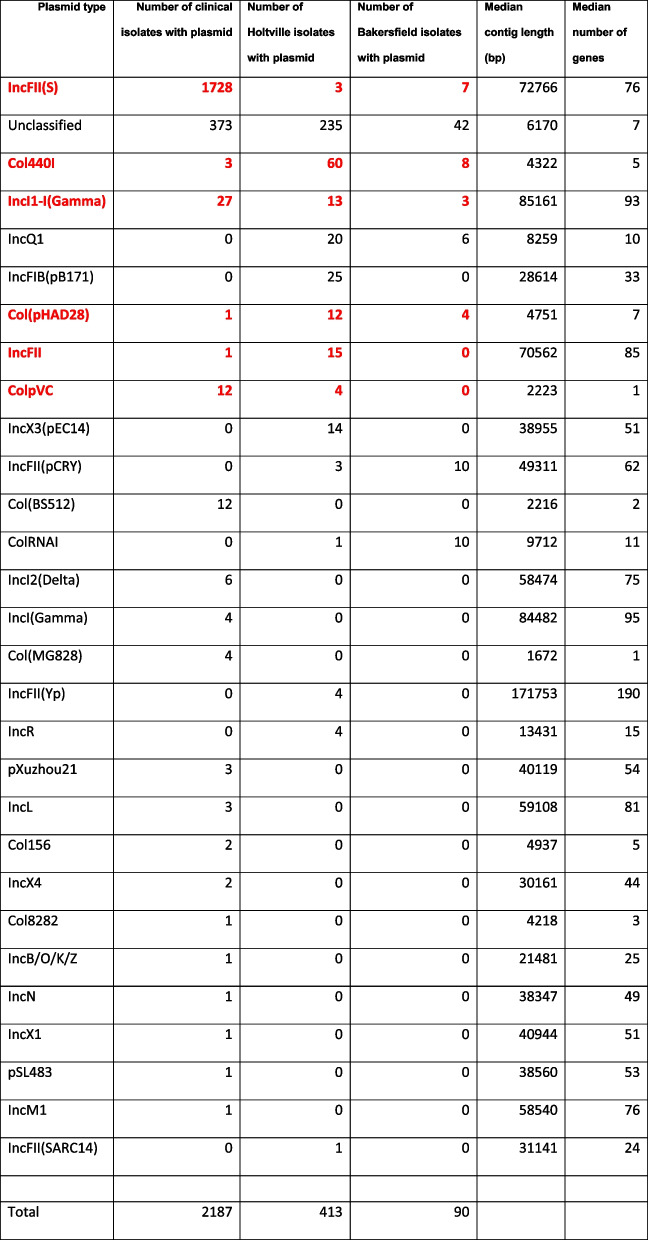
Summary of the plasmids found in the clinical and farm isolates. Plasmid types observed in both the clinical and farm isolates are bolded and in red

The 512 farm isolates, from 30 different *Salmonella* serovars, contained 227 putative extrachromosomal plasmid contigs belonging to 14 known plasmid types and 277 putative plasmid contigs of unknown type (Table 1). No plasmid type was common to all isolates and 346 isolates had no identified plasmid contigs. The most commonly observed plasmid types were the Col440I (68 isolates) and the IncQ1 (26 isolates).

Six plasmid types were observed in both the clinical and farm isolates: IncFII(S), IncFII, IncI1-I(Gamma), ColpVC, Col440I, and Col(pHAD28). There were also many uncharacterized plasmids observed in the clinical and farm isolates.

### Analysis of plasmids sharing high similarity in the the clinical and farm isolates

**Fig. 2:**
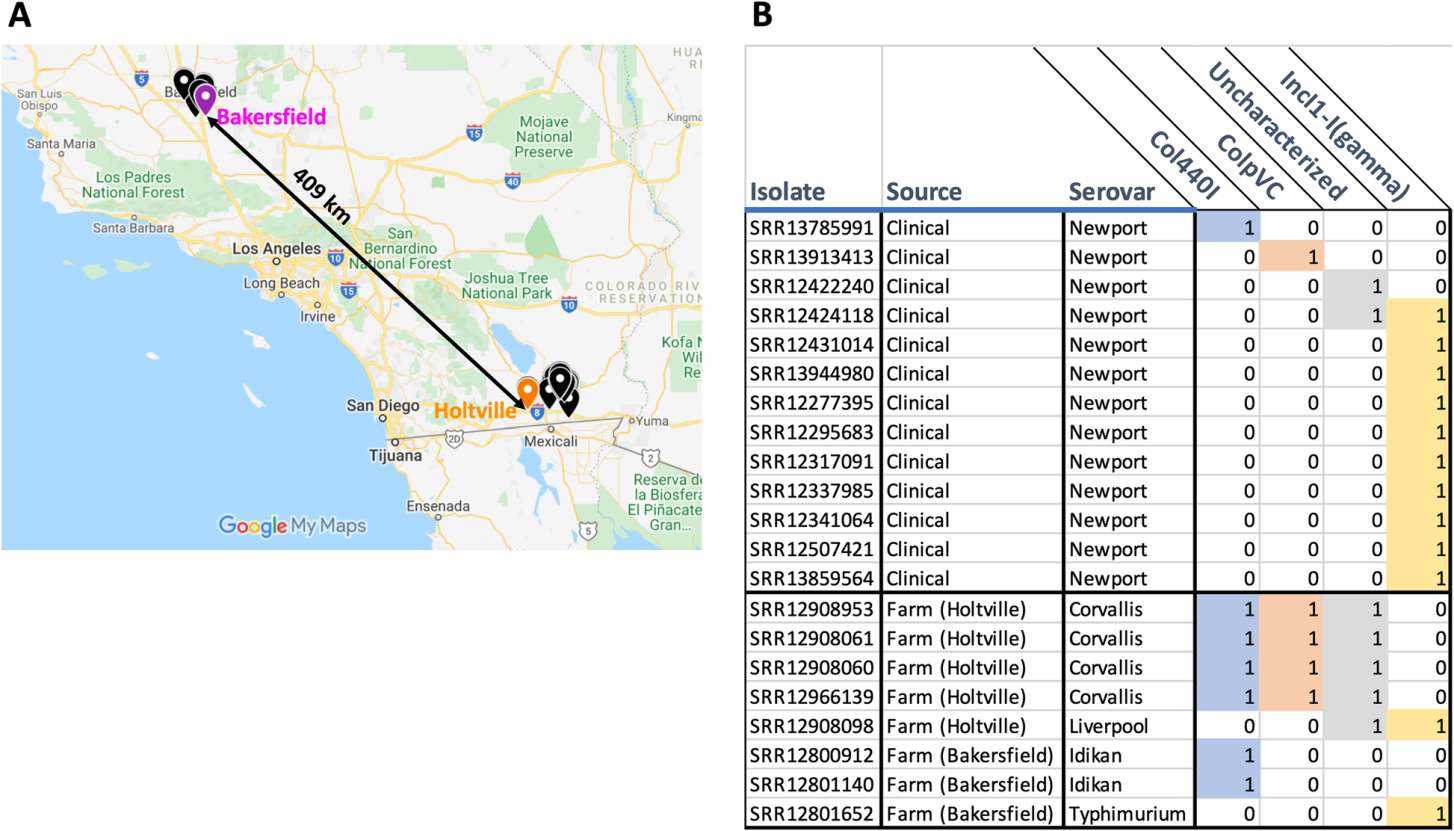
High similarity plasmids shared by the farm isolates and the clinical clade. **A** The map (made in Google My Maps) shows the 49 sampling sites where the farm isolates were collected near the Holtville and Bakersfield farm regions. The orange and magenta markers indicate the two sites, near Holtville and Bakersfield, respectively, where the highly similar plasmids were found. Most sampling sites were within 2 km of the farms, but some were up to 40 km away. **B** The table shows the clinical and farm isolates that share high similarity plasmids. Some isolates carry multiple of the high similarity plasmids e.g., SRR12908953 carries Col440I, ColpVC, and uncharacterized plasmid types

For our analysis, we sought to identify plasmids that might have undergone recent horizontal transfer between the clinical and farm isolates. To do this, we restricted our analysis to clinical and farm plasmids sharing high similarity (≥ 95% identity and 90% alignment coverage) i.e., those more likely to be related by recent horizontal transfer. Amongst the six plasmid types and the uncharacterized plasmids observed in both the clinical and farm isolates, there were 14 plasmids from 13 clinical isolates with high similarity to 17 plasmids from 8 farm isolates (Fig. 2). The highly similar clinical and farm plasmids belonged to the Col440I, ColpVC, and IncI1-I(Gamma) plasmid types as well as one uncharacterized plasmid type (Table 2). The eight farm isolates with these plasmids had been collected from 2 sampling sites separated by 409 km (Fig. 2): 1) water samples from the New River in Seeley, California (about 30 km West of Holtville); 2) soil samples near an irrigation filling station next to the Bakersfield onion farm. Five isolates (four *Salmonella* Corvallis and one *Salmonella* Liverpool) were collected from the first site and each of them carried two or three high similarity plasmids. The other three isolates, two from *Salmonella* Idikan and one from *Salmonella* Typhimurium were collected from the second site and only carried one high similarity plasmid per isolate. Amongst the clinical isolates, all carried a single plasmid with high similarity to a farm plasmid except isolate SRR12424118 which carried two.

**Table 2:** Summary of metadata for farm and best BLAST hit NCBI plasmids with high similarity (≥ 95% identity and ≥ 90% alignment coverage) to a clinical plasmid. Only NCBI plasmids from environmental sources were included. The asterisk for the IncFII(S) plasmid indicates an exception where the pMLST genes were identical between the clinical and farm plasmids, but the percentage of shared gene cargo was less than 40%. Here, phylogenetic neighborhood refers to all NCBI Pathogen Detection isolates within 1000 core gene alleles of the clinical clade

Plasmid type	Number of isolates with plasmid (clinical, Holtville, Bakersfield)			Number of highly similar plasmids (clinical, Holtville, Bakersfield)			Number of best hits in NCBI Pathogen and Nucleotide databases	Geographic extent of NCBI best hits	Environmental isolation sources of NCBI best hits	Temporal range of NCBI best hits	Bacterial host range (genera) of NCBI best hits
IncFII(S)*	1728	3	7	1728	3*	7*	30	United States, Mexico	River water, soil, almonds, pistachios, chicken	2010 to 2020	*Salmonella*
IncI1-I(Gamma)	27	13	3	10	1	1	50	United States, South Korea, Canada, United Kingdom, Denmark, Switzerland	Cow, sheep, dog, pig, chicken, catfish, horse, iguana, lettuce, soil	2002 to 2022	*Escherichia*, *Salmonella*, *Shigella*
ColpVC	12	3	0	1	4	0	100	United States, Canada	Chicken, turkey, pig	2007 to 2020	*Salmonella*
Col440I	3	60	8	1	4	2	19	United States, Germany, Mexico, Venezuela, Ecuador	Chicken, cow, pig, papaya, river water	2013 to 2020	*Escherichia*, *Salmonella*
Unclassified	373	235	42	2	5	0	12	United States, Australia, United Kingdom, India, Japan	Dog, cow, pig, chicken, wastewater	2014 to 2021	*Escherichia*, *Salmonella*, *Klebsiella*

Next, we sought to include closely related plasmids from the NCBI for phylogenetic analysis (Table 2). The 14 clinical plasmids sharing high similarity with the 17 farm plasmids were used to recruit the best basic local alignment search tool (BLAST) hits from the NCBI Pathogen Detection and Nucleotide databases. The BLAST hits were filtered for those of environmental origin and that had as high of a sequence similarity with the clinical plasmids as that between the highly similar clinical and corresponding farm plasmids. We restricted our analysis to environmental isolates to increase the chance of discovering signal from geographic and isolation sources. With the clinical, farm, and NCBI plasmid sequences, multiple sequence alignments were built, and phylogenetic analysis was performed. In some cases, the phylogenies lacked resolution, i.e., formed large polytomies, and so graphs (referred to here as mutation graphs) were built to visualize the mutational similarities and differences between the plasmid sequences. It is important to note that mutation graphs are only meant to visualize mutational differences and are not phylogenetic hypotheses [25].

For the phylogenetic analysis of the plasmids, we hypothesized that clinical and farm plasmids related by recent horizontal transfer would belong to the same subclade within the phylogeny. However, it was uncertain how close they would cluster in the phylogeny because plasmids can be rapidly transmitted between species and the transmission chain between clinical and farm isolates might have involved intermediate species [26–28]. A brief description of each plasmid as well as the results from the multiple sequence alignments, phylogenies, and mutation graphs are described in the paragraphs below.

#### IncI1-I(gamma) plasmid

IncI1 plasmids have been widely observed in *Enterobacteriaceae* and isolated from many animals. This plasmid type often confers antimicrobial resistance and/or colicin production [29]. Those analyzed here had a mean length of 85 kbp and 90 predicted genes. Most of the genes could not be functionally annotated (72 genes), however, genes with predicted functions were involved with the Type II secretion system, plasmid segregation and partitioning, bacterial outer membrane adhesion, pilus formation, DNA polymerase IV, and a Colicin-Ia operon.

The IncI1-I(gamma) plasmid type occurred in 27 clinical isolates and 16 farm isolates, but only ten clinical and two farm plasmids (1 Holtville *Salmonella* Liverpool isolate and 1 Bakersfield *Salmonella* Typhimurium isolate) shared high similarity. The pangenome of the clinical and farm plasmids sharing high similarity contained 147 genes with 69 core genes occurring in all. The whole plasmids could not be aligned due to differences in gene content and order, so the concatenated core genes were used for the multiple sequence alignment. Analysis of the multiple sequence alignment revealed that the Bakersfield and Holtville plasmids were more distant from each other (276 SNPs) than to any clinical plasmid. Further, the clinical plasmids were more similar to the Holtville (median = 162 SNPs) than the Bakersfield (median = 230 SNPs) plasmids. Additionally, the median SNP distance between the clinical isolates (146 SNPs) was similar to the distance between the clinical and Holtville farm plasmids (155 SNPs). According to metadata held internally by the FDA, the clinical isolates carrying these plasmids had been collected from patients over the duration of a month in multiple states in the USA as well as Canada.

**Fig. 3:**
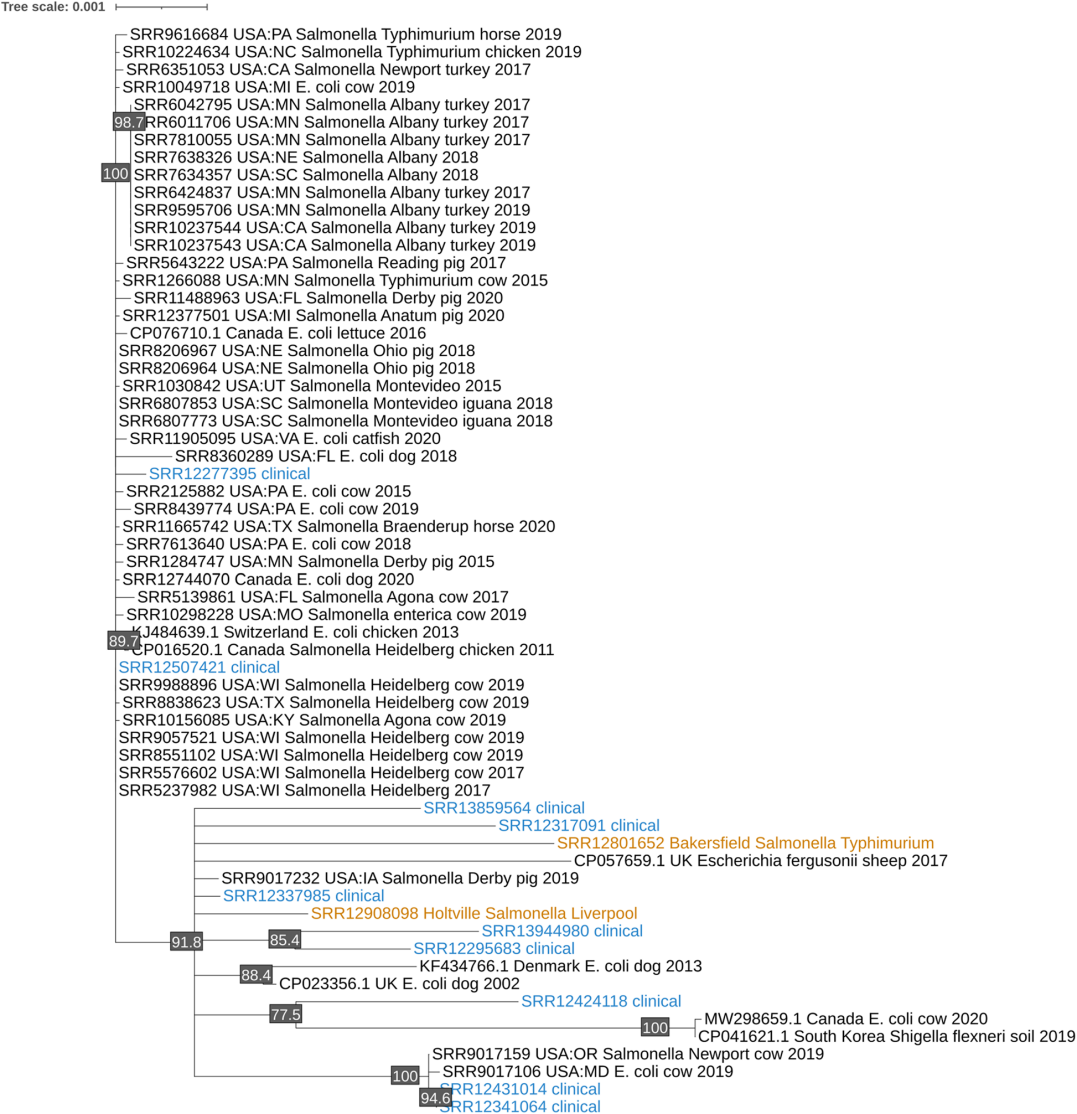
The unrooted maximum likelihood phylogeny of the IncI1-I(Gamma) plasmid. Internal nodes with less than 75% bootstrap support—1,000 bootstraps—were collapsed. The phylogeny was built from the multiple sequence alignment of the concatenated core genes (33 genes and 24,173 bp). There were 23,180 invariant sites in the multiple sequence alignment. The bootstrap values are provided as internal node labels. Of the 27 clinical isolates and 16 farm isolates that had this plasmid, only ten clinical isolates (blue) and two farm isolates (orange) shared high similarity

The pangenome of the clinical and farm plasmids and the best BLAST hits from the NCBI contained 286 genes with 28 core genes. The core gene phylogeny of these plasmids showed that eight of the clinical plasmids belonged to a subclade with the two farm isolates (Fig. 3). This subclade also contained plasmids from four *Escherichia coli*, one *Escherichia fergusonii*, one *Shigella flexneri*, one *Salmonella* Newport, and one *Salmonella* Derby isolates. These isolates were collected from 2002 to 2019, from the United States, Canada, the United Kingdom, South Korea, and Denmark, from cows, sheep, dogs, pigs, and soil samples.

The tree created from hierarchical clustering of the pangenome gene presence and absence matrix had similar groupings as the core gene phylogeny, but with more branching and a slightly different topology (Supplementary Fig. 1). All but one of the plasmids from the NCBI that had grouped in the subclade with the clinical and farm plasmids in the core gene phylogeny also grouped in the hierarchically clustered tree. However, unlike the core gene phylogeny, the hierarchically clustered tree split the Holtville and Bakersfield plasmids into separate groupings, each with their own sets of clinical plasmids.

#### Uncharacterized plasmid

There were 373 and 277 putative plasmid contigs that could not be classified as a known plasmid type in the clinical and farm isolates, respectively. Amongst these, two clinical and five farm plasmid sequences shared high sequence similarity. These sequences corresponded to a cryptic plasmid previously observed in species across the *Enterobacteriaceae* family [30–32]. The sequences analyzed here were approximately 4,200 bp long with six predicted genes including a *repB* replication gene, a *mobQ* relaxase, a conjugal transfer gene, and three hypothetical genes.

The two clinical plasmids differed by 6 SNPs and had been collected from two patients in Oregon and Missouri separated by 13 days. All five of the farm plasmids were collected on the same day at the Holtville collection site, with four in *Salmonella* Corvallis isolates and one in a *Salmonella* Liverpool isolate. The four Holtville *Salmonella* Corvallis plasmids differed from each other by 2 SNPs and the *Salmonella* Liverpool plasmid by 62 SNPs. Twelve highly similar plasmids were found in the NCBI Nucleotide and Pathogen databases for which there was no consistent name or type.

**Fig. 4:**
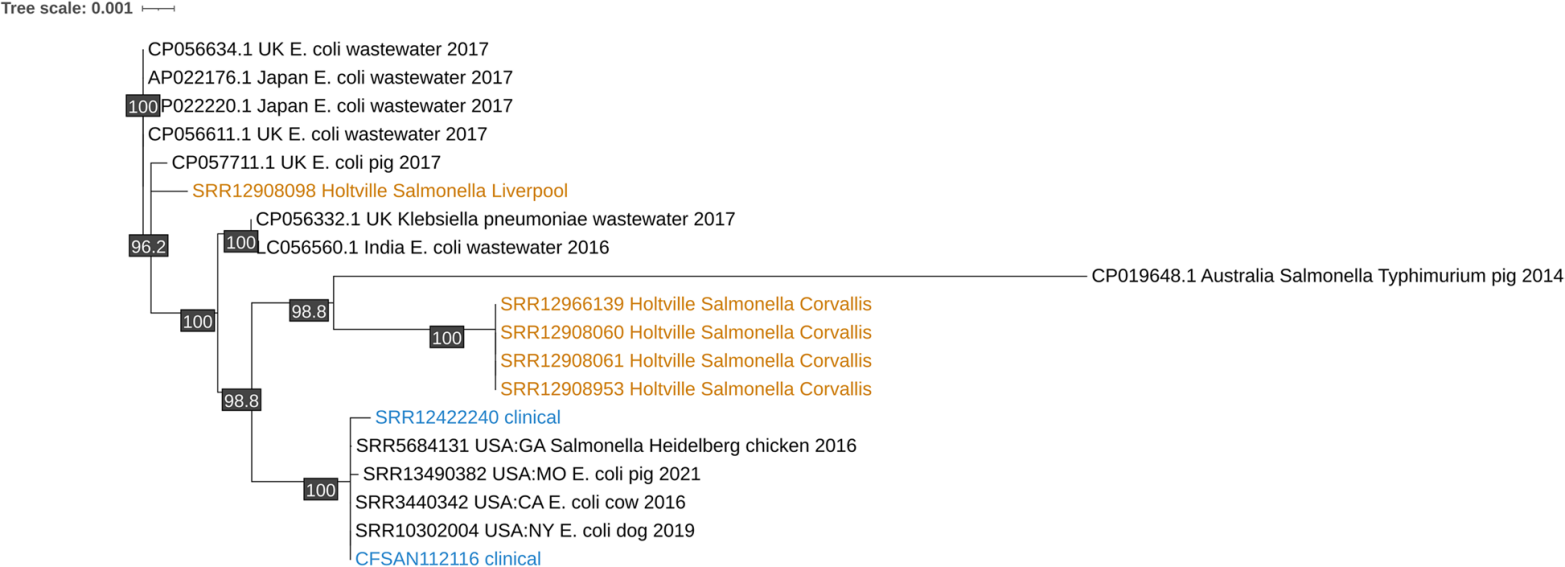
The unrooted maximum likelihood phylogeny of the uncharacterized plasmid. Internal nodes with less than 75% bootstrap support—1,000 bootstraps—were collapsed. The phylogeny was built using the multiple sequence alignment of the whole plasmids (4,201 bp) which had 3,956 invariant sites. The Holtville isolates are in orange and the clinical isolates are in blue. Four of five Holtville isolates formed a clade, each differing by 2 SNPs from the rest. These Holtville isolates were 2 SNPs different from a *Salmonella* Typhimurium isolate collected from a pig in Australia in 2014. The clinical plasmids (which differed from each other by 6 SNPs) were in a sister clade to the four Holtville plasmids, differing by 29 to 41 SNPs. Bootstrap values are provided as internal node labels

The phylogeny showed that the two clinical plasmids and four Holtville *Salmonella* Corvallis plasmids belonged to sister subclades within a larger subclade. These clinical and farm plasmids differed by 29 to 41 SNPs (Fig. 4). The subclade of the clinical plasmids also contained plasmids from three *E. coli* and one *Salmonella* Heidelberg isolates collected from several animals (dog, cow, pig, chicken) in the United States between 2016 to 2021. The subclade of the four Holtville plasmids contained one *Salmonella* Typhimurium isolate collected from a pig in Australia in 2014.

#### Col440I plasmid

The Col440I plasmid has been observed in species across the *Enterobacteriaceae* family [30, 31] and those analyzed here had three predicted genes: *qnrB* (quinolone resistance gene [33]), a phage shock protein transcription activator (*psp* gene), and a pentapeptide repeat protein. The plasmid type was only found in three clinical isolates but was the most frequently observed plasmid type in the farm isolates, occurring in 68. However, only one clinical and six farm plasmids shared high similarity. For the highly similar farm plasmids, four were found in *Salmonella* Corvallis isolates at the collection site near the Holtville farm and two were found in *Salmonella* Idikan isolates at the collection site near the Bakersfield farm.

In the NCBI Nucleotide and Pathogen databases there were 19 highly similar plasmids. These had been collected from 2013 to 2020 from diverse isolation sources (chickens, cows, pigs, papaya, river water, wastewater) and geographic locations (USA, Germany, Canada, Mexico, Venezuela, Ecuador), as well as multiple *Salmonella enterica* serovars and *E. coli*.

The phylogeny formed a single, unresolved polytomy so a mutation graph was made to visualize the differences between the sequences (Supplementary Fig. 2). The mutation graph showed that there were only 3 SNP differences between the clinical plasmid and the other plasmids, which were all identical.

#### ColpVC plasmid

The ColpVC plasmid is a cryptic plasmid [34] that has been observed in species across the *Enterobacteriaceae* family [30, 31]. Those analyzed here had one replication gene (pfam01446) and one hypothetical gene. The plasmid type was observed in 12 clinical isolates and 4 *Salmonella* Corvallis farm isolates from the Holtville collection site. Only one of the clinical ColpVC plasmids was highly similar to the 4 farm plasmids. The clinical plasmid differed by 1 or 3 SNPs from the farm plasmids. The four farm plasmids had been collected on the same day, from the same site and serovar, but differed by 0 to 4 SNPs.

Within the NCBI Nucleotide and Pathogen databases there were 100 highly similar plasmids found. These had been collected from the USA, UK, and Canada from 1999 to 2021 and were mostly from chickens and turkeys. These were mainly from *Salmonella enterica* serovars Reading, Kentucky, and Enteritidis, as well as *E. coli*.

The phylogeny formed a single, unresolved polytomy so a mutation graph was made to visualize the differences between the sequences (Supplementary Fig. 3). The mutation graph revealed 57 plasmids that were identical to the clinical plasmid. These had been collected from the USA between 2007 and 2020, from various *Salmonella enterica* serovars (mostly Reading, Kentucky, and Enteritidis), and were mainly from chickens or turkeys. None of the plasmids from the NCBI were identical to the farm plasmids, but several shared one or two of the same SNP differences with the clinical plasmid*.*

### Analysis of the IncFII(S) plasmid

The IncFII(S) plasmid was the only plasmid type carried by all clinical isolates. It was predicted to be host restricted to the *Salmonella* genus [30, 31]. The clinical IncFII(S) plasmids were approximately 73 kbp long with 77 genes. The genes coded for a Type-F conjugative transfer system, plasmid replication and persistence (e.g., *ccdA*/*ccdB* toxin-antitoxin system), as well as a *saf* fimbrial operon which is strongly correlated with increased virulence in humans [35]. Only two annotated genes (ammonia monooxygenase and succinate dehydrogenase flavoprotein) appeared to confer metabolic functions to the bacterial host.

The IncFII(S) had no full-length, high similarity matches between the clinical and farm plasmids. However, within the NCBI Pathogen Detection and Nucleotide databases, there were 30 full length, highly similar instances found in environmental isolates (25 *Salmonella* Newport and 5 *Salmonella* Javiana). The pangenome contained 133 genes with 41 core genes. The 5 *Salmonella* Javiana isolates had been collected from leafy greens and poultry—the only connection to an animal source. The 30 environmental isolates were all from California or Mexico, except one from Arizona and two from Washington. Amongst the environmental isolates, 20 had been previously identified by the whole genome SNP analysis as highly related to the clinical clade (8 to 39 SNPs different).

The core gene phylogeny of the IncFII(S) plasmid mostly formed a single unresolved polytomy except for three isolates which branched into another polytomy. As such, a mutation graph was constructed to better observe the differences between the isolates (Supplementary Fig. 4). The most similar plasmids (having one or zero SNP difference) were from the same California isolates that had formed a subclade with the clinical isolates in the SNP phylogeny.

Because the IncFII(S) plasmid type was carried by all clinical isolates and was identified in environmental isolates from the whole genome SNP analysis, we again searched the farm isolates for it. However rather than looking for full-length, high similarity matches, we only used the 3 gene pMLST profile of the clinical plasmids (FIC_5, FIIS_1, FIIY_10). This revealed 10 farm isolates with identical pMLST profiles. Three were *Salmonella* arizonae isolates collected near the Holtville farm. These three isolates shared 29 pangenome genes (≥ 95% identity) with the clinical plasmid—for reference, the median number of genes per clinical plasmid was 77. The other seven were *Salmonella* Typhimurium isolates collected from Bakersfield. These seven only shared four pangenome genes (≥ 95% identity) with the clinical plasmid.

### Assessing the significance of observing the highly similar plasmids

We suspected that the number of highly similar plasmids in the clinical and farm isolates (Table 2) was due to originating from the same regional microbiota. We sought to test our suspicion through a resampling experiment. The null hypothesis for the experiment was that just as many high similarity ColpVC, Col440I, and IncI1-I(Gamma) plasmids could be found in randomly sampled environmental *Salmonella enterica* isolates (collected across the United States and internationally) as were found in the farm isolates. Note, for this analysis the uncharacterized plasmid type was excluded because without its type we could not confidently identify all instances in the clinical and farm isolates.

**Fig. 5:**
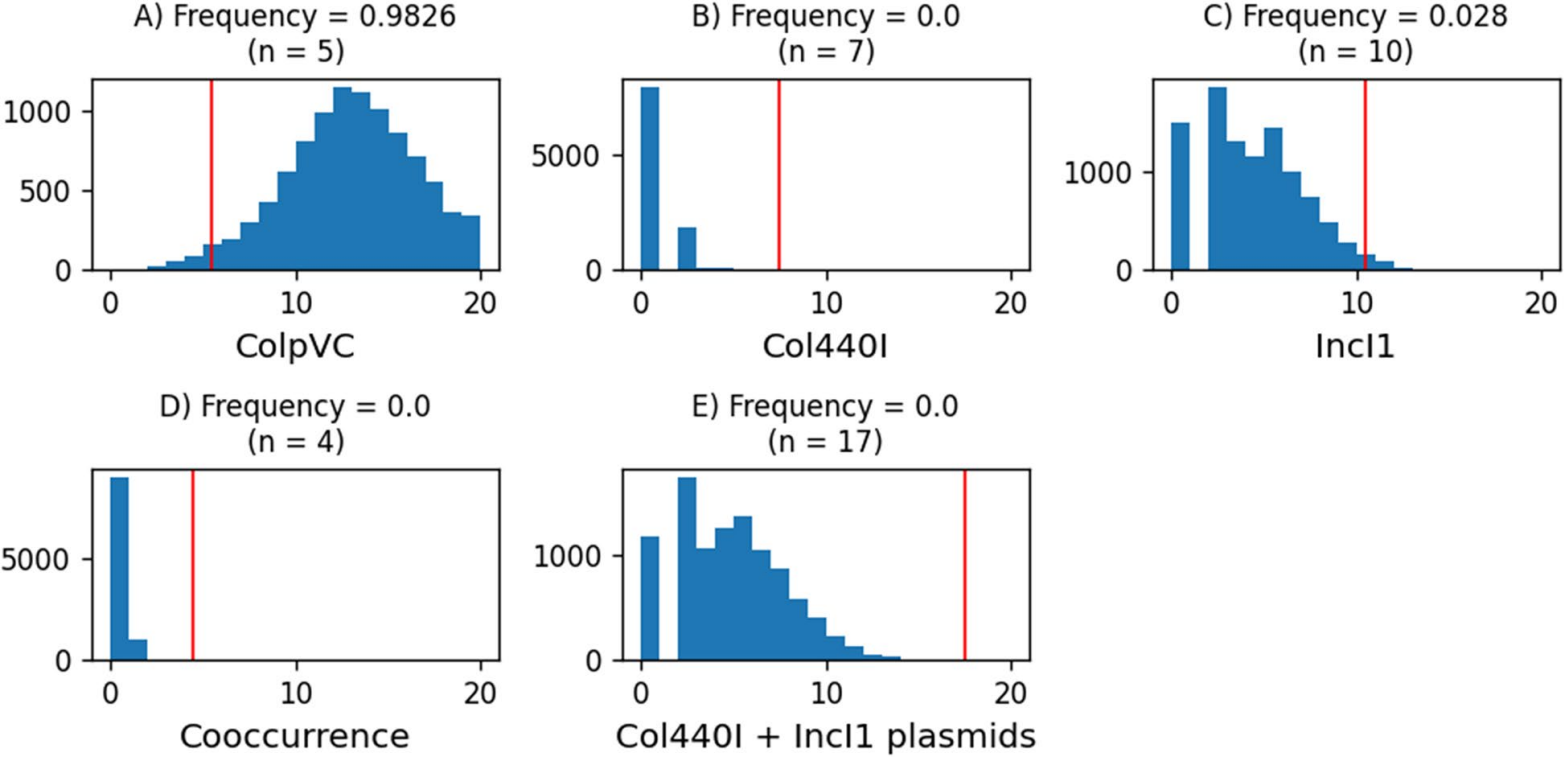
Frequency of observing highly similar plasmids in 10,000 random samples of 516 environmental *Salmonella enterica* isolates. The histograms show the number of **A** highly similar ColpVC plasmids; **B** highly similar Col440I plasmids; **C** highly similar IncI1-I(Gamma) plasmids; **D** isolates where multiple highly similar plasmids cooccur; **E** the sum of highly similar Col440I and IncI1-I(Gamma) plasmids. The red vertical lines and the value of n in the parentheses are the actual counts observed in the outbreak. Frequencies show the proportion of 10,000 samples with at least the same number of occurrences as the outbreak

The resampling experiment revealed the frequency of observing high similarity matches to clinical plasmids in random samples of environmental *Salmonella* isolates (Fig. 5). The frequency of observing the outbreak counts or higher for individual plasmid types was very high for the ColpVC plasmid type (98.3%), but 0% for the IncI1-I(Gamma) and Col440I plasmid types (Fig. 5 A-C). The phenomena of isolates simultaneously carrying two or more highly similar plasmids was never observed (Fig. 5D); for reference, 4 farm isolates from the outbreak simultaneously carried highly similar ColpVC and Col440I plasmids (Fig. 2B).

The number of highly similar ColpVC, Col440I, and IncI1-I(Gamma) plasmids observed in the outbreak was never observed in the randomly sampled environmental isolates. There was a low frequency (0.6%) of observing two of each plasmid type. But the frequency of observing a similar sum (*n* = 17) of Col440I and IncI1-I(Gamma) plasmids as in the outbreak was 0% (Fig. 5E). Here, the ColpVC plasmids were excluded because they were nearly always observed and, thus, not informative.

The low frequency of observing Col440I and IncI1-I(Gamma) plasmids, with as high of sequence similarity as between the clinical and farm plasmids, in random samples of environmental *Salmonella enterica* isolates led us to reject our null hypothesis.

## Discussion

The epidemiological investigation of the 2020 *Salmonella* Newport onion outbreak strongly implicated two onion farms in Holtville and Bakersfield California as the likely source of the outbreak [5, 7]. Our SNP analysis supported that the clinical isolates likely originated from central California because the most closely related environmental isolates in the NCBI Pathogen Detection database were mainly collected from California and almonds; nearly 100% of the almonds grown in the United states come from California and ~ 75% are produced in five counties (Stanislaus, Fresno, Kern, Merced, and Madera) in central California including where Bakersfield is located [36, 37].

The outbreak strain was never recovered from the Holtville and Bakersfield farm regions. Nonetheless, we hypothesized that if the outbreak strain had come from the farm regions it might carry horizontally transferred plasmids acquired from the local microbiota—here, represented by the farm isolates. We identified highly similar plasmids in the clinical and farm isolates but the apparent promiscuity of the plasmids (e.g., isolated from multiple species within *Enterobacteriaceae*, isolated from multiple animal and environmental sources, an international distribution, all within the last 20 years) prevented the extraction of information from the phylogenies about geographic location, isolation source, the time of transfer, and if there had been direct transfer between the clinical and farm *Salmonella* serovars or if there were intermediary species. The construction and dating of plasmid phylogenies is especially complex because plasmids, as units, can be vertically and horizontally transmitted, and their parts can undergo extensive recombination, including horizontal transfer [38]. In particular, state-of-the-art methods assume (1) that the set of genes used in the concatenated multiple sequence alignment share a common evolutionary history and (2) that the rate of substitutions is clock-like (e.g., the least square dating method implemented in IQ-Tree 2 assumes a relaxed molecular clock model [39]) [40–42]. For plasmids, these assumptions can be violated if individual genes were horizontally acquired from different sources and thus have different evolutionary histories where they were subjected to different selection pressures, leading to different substitution rates [31, 38, 43].

Although promiscuity limited the conclusions drawn from individual plasmid phylogenies, we argue that the number and combination of highly similar plasmids carried by an outbreak strain and microbiota from a suspected environmental source is potentially a unique and valuable epidemiological marker. Our resampling analysis indicated it was unlikely to observe the number and combination of highly similar plasmids in random samples of environmental *Salmonella enterica*—evidence that the outbreak strain had undergone horizontal transfer with the microbiota from the implicated farm regions. Underlying our resampling analysis is the hypothesis that the number of highly similar plasmids shared by isolates should decrease as geographic distance increases. The dataset used for our case study and the scope of our analysis was not sufficient to test this hypothesis because the isolates were only collected from sick patients and the farm regions. Future work should test this hypothesis on other datasets to understand if there is a correlation between the number of shared plasmids and geographic distance, at what geographic distances it is applicable, to quantify the expected numbers of shared plasmids, if it varies by plasmid type, etc. Additionally, studies should continue to assess the burden of evidence needed to relate a pathogen to a geographic location using horizontally transferred elements.

An important consideration highlighted by our analysis, is that the relevant plasmids were rare in the clinical and farm isolates—thousands of isolates were necessary to identify them. Therefore, future studies should examine large sets of isolates. A limitation of our study was that the *Salmonella enterica* isolates, collected during the on-site investigation of the farms, were the only representatives of the farm microbiome. Although plasmids can be directly transferred between *Salmonella enterica* serovars, it is unclear how frequently that occurs in the environment. Also, because conjugation is dependent upon the proximity and density of donor and recipient cells, the local microbiota often serves as a reservoir for plasmids and as an important intermediary [26–28]. Therefore, the whole environmental microbiome should have been investigated to better understand the presence of the plasmids and to increase the chance of detecting horizontally transferred plasmids. It also would have been informative to sample the gut microbiomes of local animals like cattle, which are thought to be a reservoir of *Salmonella* Newport [2], because the gut microbiome presents ideal conditions for the horizontal transfer and persistence of plasmids [26–28]. Additionally, the gut microbiomes of patients should have been sampled to help differentiate plasmids that were acquired there from other sources. These considerations point out some of the complexities of sampling adequately to understand plasmids observed in outbreak isolates.

Identifying the 20 plasmid types in the nearly-clonal clinical clade was surprising given that our literature review only found 11 plasmid types that had been previously observed in *Salmonella* Newport [15–21]. This indicates a much greater diversity of plasmids in *Salmonella* Newport than currently reported and warrants further investigation. That all but one of the plasmid types occurred at low frequency in the clinical isolates suggests recent acquisition via horizontal transfer and might be explained by the dynamics of the outbreak, e.g., a population that rapidly grew and changed environments. Horizontal transfer can increase as a result of transitioning from the environment, or animal reservoir, to humans, such as might happen to pathogens that cause a foodborne illness outbreak [44]. Future work should further explore how outbreaks affect plasmid diversity within pathogen populations. Additionally, although we focused on plasmids because they constituted most of the accessory genome, future studies should explore other types of mobile genetic elements such as phages and transposons.

The analysis of the plasmid multiple sequence alignments, phylogenies, and mutation graphs highlighted important gaps in our knowledge about specific plasmid types—especially at very granular levels of microevolution. For example, identical instances of the clinical ColpVC plasmid (Supplementary Fig. 3) could be found over the last 15 years. And yet the farm ColpVC plasmids differed by 0 to 4 SNPs despite being collected from the same sampling site on the same day. Another example is the uncharacterized plasmid, where the two clinical instances differed by 6 SNPs (collected 13 days apart from different patients in different states) and were grouped with environmental isolates which had been collected up to six years before (Fig. 4). Perhaps the clinical instances represent different lineages of the plasmid and were independently acquired, but even the plasmid instances carried by the four *Salmonella* Corvallis farm isolates—collected from the same sampling site on the same day—differed from each other by two SNPs. An alternative explanation is plasmid heterozygosity in the clinical and farm isolates i.e., copies of a plasmid with sequence variations within a single cell or population [45]. It is also possible that the plasmids might have a high mutation rate but the rate at which they are fixed in the population is low [46]. Adding further complexity, the *Salmonella* Liverpool instance of the uncharacterized plasmid differed from the *Salmonella* Corvallis instances by 62 SNPs despite being collected from the same sampling site and the same day, indicating potential barriers to conjugation despite proximity.

Our work also stresses the need for improving the comprehensiveness of plasmid classification. Commonly used plasmid typing methods (e.g., MOB and incompatibility typing) can fail to classify 50% of the plasmids in highly curated datasets (e.g., RefSeq plasmids) much less environmental samples [47]. Further, current typing methods are most comprehensive for *Enterobacteriaceae,* but we still observed hundreds of putative plasmid contigs that could not be classified. Related to classification, it can be difficult to assess the evolutionary relatedness of plasmid sequences [47, 48]. For example, the three pMLST genes of the IncFII(S) plasmid were identical in the clinical isolates as well as several farm and environmental isolates going back a decade. When using the replicon-based pMLST system [49], these plasmids would be considered highly related. However, the amount of shared gene cargo for these plasmids ranged from 5 to 100%—in all cases, the shared gene cargo had high sequence similarity. Here, using the proportion of shared genes as a distance metric [48] would find the plasmids with less shared gene cargo as more divergent. Methods are needed to reconcile the molecular evolution of core genes and cargo genes when analyzing plasmids.

Together, these issues underscore that more work is needed to characterize the molecular evolution of specific plasmid types. Additionally, more environmental sampling is needed to better understand the distribution, population dynamics, and transmission dynamics of plasmids in the environment. Essential to this effort are comprehensive sequence databases and quality metadata. Additional sequence data and metadata might have revealed more details about the plasmid phylogenies e.g., the transmission chain between bacterial species, the geographic source, the isolation source, timeframe of transmission. On a cautionary note, it is important that genome databases not remove “redundant” sequences based upon core genome similarity because the clinical clade had a large, highly conserved core genome, but it was the sparsely distributed accessory genome that made this analysis possible.

## Conclusion

The epidemiological investigation of the large 2020 *Salmonella* Newport red onion outbreak implicated two farms in California as the likely source of contamination. SNP analysis supported that the nearly-clonal clinical clade had ancestry in California going back a decade. SNP analysis also confirmed that none of the environmental *Salmonella enterica* collected from the farm regions were the outbreak strain. We explored an alternative method for analyzing the whole genome sequencing data driven by the hypothesis that if the outbreak strain had come from the farm regions, then the clinical isolates would disproportionately contain plasmids found in isolates from the farm regions due to horizontal transfer. Although the clinical clade had a large and conserved core genome it also had a large and sparsely distributed accessory genome containing more plasmid types than could be found in the literature for all *Salmonella* Newport. A small number of plasmids, 14 plasmids from 13 clinical isolates and 17 plasmids from 8 farm isolates, were found to be highly similar (95.4% to ~ 100% identical)—indicating they might be related by horizontal transfer. Phylogenetic analysis of the plasmids provided little information about their geographic origin, isolation source, or time of transfer seemingly due to promiscuous horizontal transfer. However, our resampling analysis suggested that observing a similar number and combination of highly similar plasmids in random samples of environmental *Salmonella enterica* within NCBI Pathogen Detection database was unlikely. This supported the findings of the FDA and CDC investigation that the outbreak strain had likely originated from the two farm regions in California. Horizontally transferred plasmids provided evidence for a connection between clinical isolates and the farms implicated as the source of the outbreak. Our case study suggests that such analyses might add a new dimension to source tracking investigations, but highlights the need for detailed and accurate metadata, more extensive environmental sampling, and a better understanding of plasmid molecular evolution.

## Methods

### Data and sequencing

The clinical clade consisted of 1,728 clinical *Salmonella enterica* (serovar Newport) isolates collected during the 2020 onion outbreak (June to October) in the United States (1,173 isolates) and Canada (555 isolates) (https://www.cdc.gov/salmonella/newport-07-20/index.html). The farm isolates consisted of 512 environmental *Salmonella enterica* isolates, from 30 *Salmonella enterica* serovars, collected from 49 locations, between 2020 and 2021, on and near the onion farms implicated by the epidemiological traceback as well as the nearby irrigation district and public lands. Permission was obtained by the FDA from the farm owners before collecting the environmental samples. Illumina MiSeq short read sequencing data was available for all clinical and environmental isolates (Supplementary File 1).

In addition to the short-read sequencing data, 23 isolates (15 clinical, 8 environmental) were chosen for long-read sequencing so that closed genomes could be acquired. The closed genomes were used to provide more detailed information about the genome biology of the isolates and to validate the presence of plasmids. The 15 clinical isolates were sequenced with Oxford Nanopore technology and the 8 environmental isolates were sequenced with the Pacific Bioscience (PacBio) technology (Supplementary File 1). The clinical isolates were chosen to maximize gene coverage of the pangenome (covered 74.4% of the pangenome) estimated using the 1,728 clinical short read assemblies.

The bacteria were grown overnight in tryptic soy broth (TSB) at 37˚C and genomic DNA was extracted using the Maxwell RSC cultured cell DNA kit (Promega, Madison, WI) following the manufacturer’s protocols. The DNA was used to construct libraries for long-read sequencing on the GridIon (Oxford Nanopore Technologies, Oxford, UK) using the rapid sequencing kit RBK004 and run on a MIN106D flow cell (R9.4.1) for 48 h according to the manufacturer’s instructions.

For the 8 environmental isolates, multiplexed microbial SMRTbell libraries were prepared using the SMRTbell Template Prep Kit 2.0 according to PacBio protocol “Preparing Multiplexed Microbial Libraries Using SMRTbell Express Template Prep Kit 2.0” (PacBio, Menlo Park, CA, November 2021). The multiplexed SMRTbell library was then sequenced on a PacBio Sequel IIe sequencer (PacBio, Menlo Park, CA) using Binding Kit 2.2 and Sequel II sequencing Kit 2.0 on one SMRT cell 8 M (PacBio, Menlo Park, CA), with 30 h collection time.

### Phylogenetic analysis of the clinical clade

The SNP matrix was generated by reference-based SNP analysis implemented in the CFSAN SNP Pipeline [9] with default parameters. The reference genome used for SNP analysis by this study was one of the clinical isolates from the outbreak. It was selected from the clinical isolates for having one of the highest N50’s and lowest number of contigs. The maximum likelihood phylogeny was inferred from the SNP matrix, using GARLI v2.01 [50] under the General Time-Reversible (GTR) model with Gamma distributed rate heterogeneity, estimate invariant sites, 1000 bootstraps, and 2 categories of variable rates. The phylogeny was rooted using NCBI SRA isolate SRR13685683—a *Salmonella* Newport clinical isolate collected in 2018—as the outgroup.

To explore the phylogenetic neighborhood of the clinical clade (Fig. 1C), all environmental isolates in the NCBI Pathogen Detection database that were within 1,000 core gene alleles of the clinical clade (using the 1,152 gene cgMLST scheme developed for *Salmonella enterica* at the FDA [51]) were recruited for phylogenetic analysis as described in the previous paragraph. The phylogenies were visualized with FigTree (v1.4.4) [52].

### Genome assembly, gene prediction and annotation, and pangenome estimation

The short reads were assembled with SPAdes (v3.13.0) [53] using default settings. For quality control, contigs shorter than 500 bp or with less than 10X depth of coverage were removed. Genes in the contigs were predicted and annotated with Prokka (v1.14.5) [54]. Further annotations were obtained running eggNOG-Mapper (v2.1.6) [55], using default parameters, on the predicted genes. The predicted genes were clustered with Roary (v3.12.0) [56] to identify the pangenome of the clinical clade using 90% identity for clustering and requiring that 99% of isolates possess a gene to be considered core.

The PacBio sequencing data was demultiplexed by running the Demultiplex Barcodes application and de novo assembly was done using the Microbial Assembly in SMRTLink v.10 (PacBio, Menlo Park, CA). The nanopore sequencing data, and their corresponding MiSeq short reads, were de novo assembled with Unicycler v.0.4.8 [57]. Nanopore reads shorter than 5 kbp were not used for constructing the hybrid assembly.

The long-read sequencing datasets were used to generate 23 complete genomes. These were circularized, oriented to start at the *dnaA* gene, uploaded to NCBI (Supplementary File 1), and annotated using the NCBI Prokaryotic Genome Annotation Pipeline (PGAP) v5.3.

### Identification and annotation of plasmids

Platon (v1.6) [58], using default settings, was used to identify and annotate the plasmids in the assemblies. Platon is a tool for the identification of extrachromosomal plasmid contigs in short read draft assemblies. Contigs are characterized by testing for circularization; the detection of incompatibility groups; the detection of rRNA genes; the detection of antimicrobial resistance genes; a homology search against reference plasmid sequences; the detection of oriT sequences; the detection of plasmid replication genes; the detection of mobilization genes; and the detection of conjugation genes [58]. The online COPLA server [30] was used to determine the host range of the plasmids.

The most observed plasmid type was the IncFII(S), annotated as present by Platon in 98.5% of the isolates. The IncFII(S) plasmids, identified by Platon, were BLAST aligned against the pMLST [49] database to identify the sequence profiles. To determine if the IncFII(S) plasmid was present in all clinical isolates, the raw reads of all isolates were mapped to the IncFII(S) plasmid of isolate SRR12199170 using Bowtie2 [59] with default settings. A custom Python script was used to calculate the breadth of coverage of mapped reads for each isolate. Additionally, to identify all contigs belonging to IncFII(S) in all isolate assemblies, the contigs were BLAST aligned (95% identity) to the SRR12199170 plasmid. For the 24 isolates with no identified IncFII(S) plasmid, 23 had reads mapping, with > 92% breadth of coverage, to the complete IncFII(S) plasmid in isolate SRR12199170, and one isolate had 7% breadth of coverage.

### Validation of plasmids identified in the short-read assemblies

The closed genomes that had been long read sequenced with nanopore and PacBio technologies were used to validate the presence of plasmids in the short-read assemblies. Platon was used to confirm that all the DNA sequences shorter than the chromosome in the closed genomes were plasmids. The plasmids identified in the short-read assemblies were BLAST aligned to those in the nanopore and Pacbio assemblies. Plasmids that aligned with ≥ 99% identity and ≥ 99% query coverage were counted as present. This approach confirmed the presence of all plasmids (21 in the environmental isolates and 36 in the clinical isolates) except for a IncFII(S) plasmid that only partially assembled in the short-read assembly of a clinical isolate (only ~ 10% was found in the assembly and the short reads) and a ColpVC plasmid which was not found in the short reads or assembly of an environmental isolate.

### Comparison of clinical and farm plasmids and finding the best BLAST hits in NCBI

The clinical and farm isolate plasmids were pairwise BLAST aligned (using 95% identity). The farm plasmids were filtered for those that aligned with at least 90% coverage of a clinical plasmid using a custom Python script. Metadata internal to the FDA was used to determine the GPS coordinates of the sampling locations and the *Salmonella* serovars.

Farm and clinical plasmids that BLAST aligned, were then queried against the NCBI Pathogen Detection and Nucleotide databases using BLAST (≥ 95% identity). The farm plasmids were filtered for those that aligned with at least 90% coverage of a clinical plasmid using a custom Python script. The results were additionally filtered with a custom Python script for hits that were at least as similar as the farm and clinical plasmids were to each other.

The metadata for the best BLAST hits was either parsed from the GenBank file online, if from NCBI Nucleotide database, or from the metadata provided on the NCBI Pathogen Detection website. The metadata was used to select for environmental isolates and to filter out clinical isolates so that information about geographic location and the isolation source could be analyzed.

### Plasmid phylogenies, mutation graphs, and relatedness

The matching clinical and farm plasmids, as well as the filtered BLAST hits of the clinical plasmids from the NCBI Pathogen Detection and Nucleotide databases, as described earlier, were used to build maximum likelihood phylogenies—this was also done for the IncFII(S) plasmid and its best BLAST hits, although there was no matching farm plasmid.

For the Col440I, ColpVC, and the uncharacterized plasmids (Fig. 4), a custom Python script was used to trim the sequences if they were over-circularized and to reorient the sequences to a common start locus. Then whole plasmid alignment was performed with Muscle [60].

For the IncI1-I(Gamma) and IncFII(S) plasmids (Fig. 3 and Supplementary Fig. 4, respectively), recombination, gene gain and loss, and potentially assembly errors made it difficult to perform whole plasmid alignment, so Roary was used to find the core genes (> 95% identity, 100% of isolates must have gene to be core), which were then concatenated and aligned by Roary using MAFFT (v7.305b) [61]. For the IncFII(S) plasmids, only one clinical instance was used (isolate SRR12199170) because all the clinical instances clustered at > 99% identity and > 95% coverage with MMSeqs2 [62]. Phandango [63] was used to visualize the Roary results for the IncI1-I(gamma) plasmid (Supplementary Fig. 1).

Maximum likelihood phylogenies were built from the multiple sequence alignments using IQ-Tree 2 [39] (with 1,000 bootstraps) and ModelFinder to select the substitution model [64]. Branches with less than 0.75 bootstrap support were collapsed. The phylogenies were visualized with FigTree or the Interactive Tree Of Life (iTOL) on EMBL [65].

In some cases, the phylogenies lacked resolution (formed large polytomies) and so graphs, referred to here as mutation graphs, were built to visualize the mutational similarities and differences between the plasmid sequences. Mutation graphs were constructed for the Col440I (Supplementary Fig. 2), ColpVC (Supplementary Fig. 3), and IncFII(S) (Supplementary Fig. 4) plasmids by identifying all variations in the multiple sequence alignments between the sequences and the respective clinical plasmids using a custom Python script. It is important to note that mutation graphs are only meant to visualize mutational differences and are not phylogenetic hypotheses [25].

Snippy [66] was used to determine the number of SNP differences between the uncharacterized plasmids. A custom Python script was used to measure the total number of pairwise nucleotide differences for the sequences in the concatenated core gene alignment of the IncI1-I(Gamma) plasmid. In this case, differences were measured per column of the multiple sequence alignment for every two sequence combinations. Here a nucleotide difference could be a mismatch between different nucleotides or a nucleotide and a gap—caused by an insertion or deletion.

### Statistical analysis for assessing the frequency of observing highly similar plasmids in environmental *Salmonellaent﻿erica*

We sought to assess through a resampling experiment if the number of highly similar plasmids in the clinical and farm isolates (Table 2) was due to originating from the same regional microbiome. The null hypothesis for the experiment was that just as many high similarity ColpVC, Col440I, and IncI1-I(Gamma) plasmids could be found in randomly sampled environmental *Salmonella enterica* isolates—which had been collected across the United States and internationally—as were found in the farm isolates. For this analysis the uncharacterized plasmid type was excluded because we could not confidently identify all instances in the clinical and farm isolates.

To test the null hypothesis, 516 isolates (the number of farm isolates) were randomly sampled from the 95,284 environmental *Salmonella enterica* in the NCBI Pathogen Detection database (excluding those from the outbreak). All the clinical ColpVC, Col440I, and IncI1-I(Gamma) plasmids—not just those that were highly similar to the farm isolate plasmids—were then aligned to the assemblies of the 516 isolates. Assemblies with plasmids sharing as high a sequence similarity as the highly similar clinical and farm plasmids were counted (Supplementary Table 1). This was repeated 10,000 times and the frequency of observing the plasmids was recorded.

The metadata for *Salmonella enterica* was downloaded from the NCBI Pathogen Detection ftp site on March 28, 2022. A custom Python script was used to randomly select 516 environmental isolates. All the clinical ColpVC, Col440I, and IncI1-I(Gamma) plasmids were BLAST aligned to the 516 assemblies of the randomly selected environmental isolates. A custom Python script was used to parse the BLAST results. To calculate the percent identity of all local alignments to a clinical plasmid, the clinical plasmid was initially represented as an array of zeros. Every position within the clinical plasmid was updated with the maximum percent identity from all local alignments. If the average percent identity of the array representing the alignment to the clinical plasmid was as high as the minimum percent identity for the relevant plasmid type (Supplementary Table 1), it was counted as a highly similar. Random sampling of the environmental isolates and finding highly similar plasmids was repeated 10,000 times to build distributions. The distributions were used to assess the frequency of observing the highly similar plasmids. A custom Python script was used to visualize the results.

## Supplementary Information

**Additional file 1: Supplementary File 1.** Is an excel spreadsheet containing all of the NCBI SRA accessions of the clinical, farm, and environmental isolates used for our analysis. Each category of isolate (i.e., clinical, farm, and environmental) is specified in the columns as well as if it was short read or long read sequenced. **Supplementary Figure 1.** IncI1-I(gamma) plasmid hierarchically clustered tree and gene presence/absence matrix. The tree and gene presence/absence matrix were generated by Roary and were visualized with Phandango. **Supplementary Figure 2.** Col440I plasmid mutation graph using the clinical plasmid as the reference. For example, A1922 means a plasmid was identical to the clinical plasmid except a mutation to A at loci 1922. The graph was built using the multiple sequence alignment of the plasmids (2,699 bp) which had 2,696 invariant sites. This graph shows mutational differences and does not necessarily represent phylogenetic relationships. **Supplementary Figure 3.** ColpVC plasmid mutation graph using the clinical plasmid as the reference. For example, G1403 means a plasmid was identical to the clinical plasmid except a mutation to G at loci 1403 (a “-“ mutation indicates a deletion). The graph was built using the multiple sequence alignment of the plasmids (2,258 bp) which had 2,237 invariant sites. This graph shows mutational differences and does not necessarily represent phylogenetic relationships. **Supplementary Figure 4.** The IncFII(S) plasmid mutation graph using the clinical plasmid as the reference. The mutation graph was built from the multiple sequence alignment of the 41 concatenated core genes (30,741 bp) which had 29,882 invariant sites. For example, C8713 indicates a plasmid was identical to the clinical plasmid except for a mutation to C at loci 8713 (a “-“ mutation indicates a deletion). This graph shows mutational differences and does not necessarily represent phylogenetic relationships. **Supplementary Table 1.** Summary of the plasmid types, with highly similar instances, observed in both the clinical and farm isolates that were used for statistical analysis. The uncharacterized plasmid was not used because all instances could not be identified in the clinical isolates. Two of the IncI1-I(Gamma) plasmids were not considered highly similar for this analysis because they did not fall into the same subclade as the farm instances.

## Data Availability

The read sets used for this analysis are all publically available from NCBI. The SRA accessions are listed in Supplementary File 1. The metadata for the isolates was downloaded and extracted from the NCBI Pathogen Detection ftp site (https://ftp.ncbi.nlm.nih.gov/pathogen/Results/Salmonella/PDG000000002.2417/Metadata/PDG000000002.2417.metadata.tsv). The reference genome used for SNP analysis by this study was one of the clinical isolates from the outbreak (NCBI SRA accession SRR12199170).
