# Supplementary material for: Assessment of plasmids for relating the 2020 Salmonella enterica serovar Newport onion outbreak to farms implicated by the outbreak investigation
Source: BMC Genomics. 2023 Apr 4;24:165. doi: 10.1186/s12864-023-09245-0 (PMC10074901; doi:10.1186/s12864-023-09245-0)
Supplement: Supplementary file 1 — Additional file 1: Supplementary File 1. Is an excel spreadsheet containing all of the NCBI SRA accessions of the clinical, farm, and environmental isolates used for our analysis. Each category of isolate (i.e., clinical, farm, and environmental) is specified in the columns as well as if it was short read or long read sequenced. Supplementary Figure 1. IncI1-I(gamma) plasmid hierarchically clustered tree and gene presence/absence matrix. The tree and gene presence/absence matrix were generated by Roary and were visualized with Phandango. Supplementary Figure 2. Col440I plasmid mutation graph using the clinical plasmid as the reference. For example, A1922 means a plasmid was identical to the clinical plasmid except a mutation to A at loci 1922. The graph was built using the multiple sequence alignment of the plasmids (2,699 bp) which had 2,696 invariant sites. This graph shows mutational differences and does not necessarily represent phylogenetic relationships. Supplementary Figure 3. ColpVC plasmid mutation graph using the clinical plasmid as the reference. For example, G1403 means a plasmid was identical to the clinical plasmid except a mutation to G at loci 1403 (a “-“ mutation indicates a deletion). The graph was built using the multiple sequence alignment of the plasmids (2,258 bp) which had 2,237 invariant sites. This graph shows mutational differences and does not necessarily represent phylogenetic relationships. Supplementary Figure 4. The IncFII(S) plasmid mutation graph using the clinical plasmid as the reference. The mutation graph was built from the multiple sequence alignment of the 41 concatenated core genes (30,741 bp) which had 29,882 invariant sites. For example, C8713 indicates a plasmid was identical to the clinical plasmid except for a mutation to C at loci 8713 (a “-“ mutation indicates a deletion). This graph shows mutational differences and does not necessarily represent phylogenetic relationships. Supplementary Table 1. Summary of the plasmid [file 12864_2023_9245_MOESM1_ESM.docx]

Supplementary File 1: Is an excel spreadsheet containing all of the NCBI SRA accessions of the clinical, farm, and environmental isolates used for our analysis. Each category of isolate (i.e., clinical, farm, and environmental) is specified in the columns as well as if it was short read or long read sequenced.


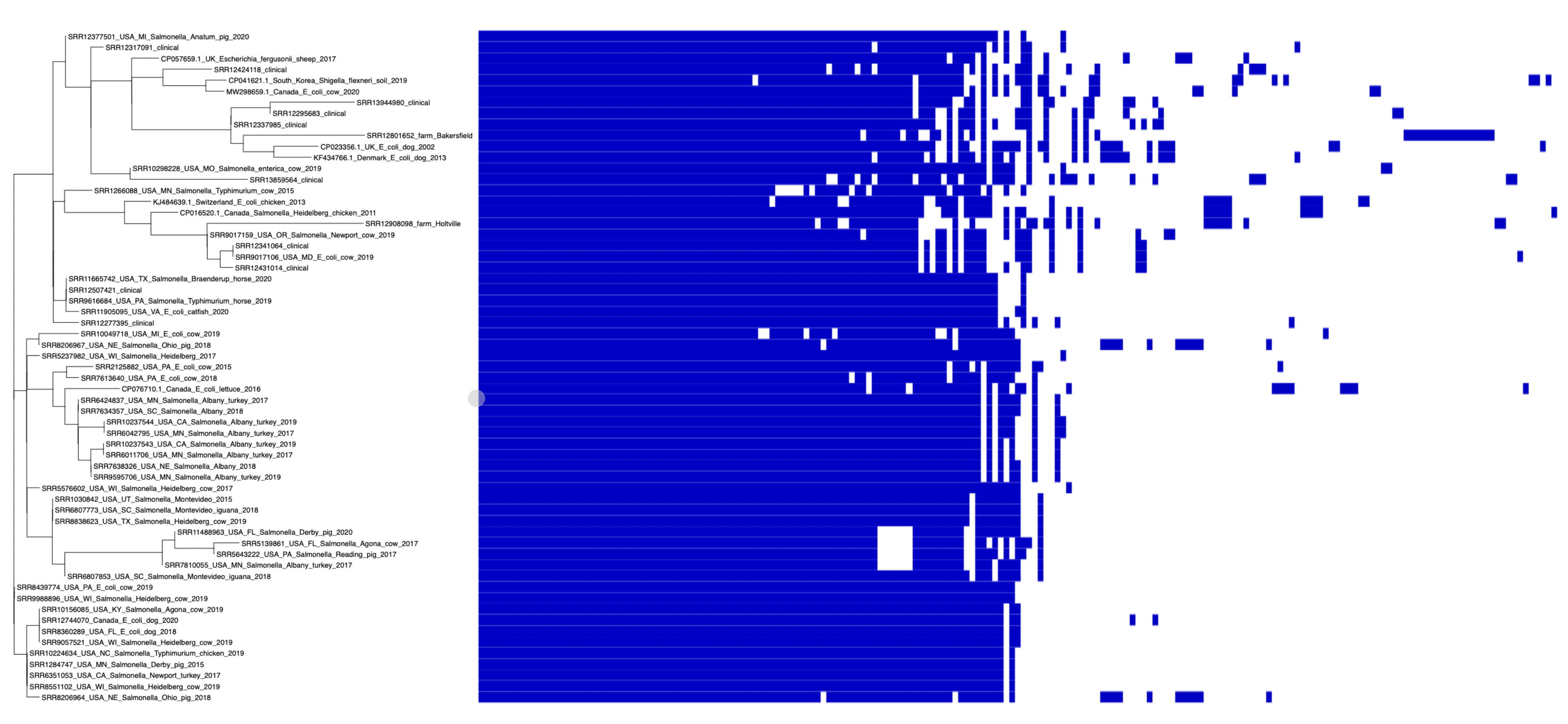


Supplementary Figure 1: IncI1-I(gamma) plasmid hierarchically clustered tree and gene presence/absence matrix. The tree and gene presence/absence matrix were generated by Roary and were visualized with Phandango.


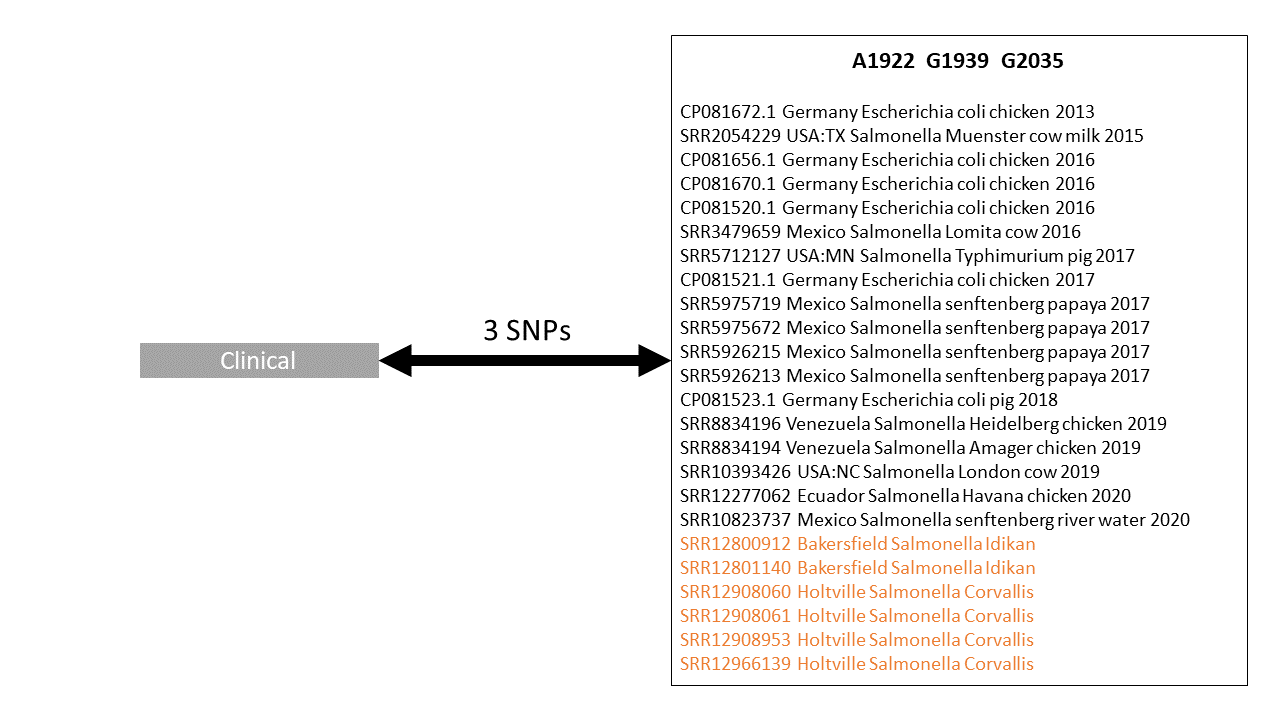


Supplementary Figure 2: Col440I plasmid mutation graph using the clinical plasmid as the reference. For example, A1922 means a plasmid was identical to the clinical plasmid except a mutation to A at loci 1922. The graph was built using the multiple sequence alignment of the plasmids (2,699 bp) which had 2,696 invariant sites. This graph shows mutational differences and does not necessarily represent phylogenetic relationships.


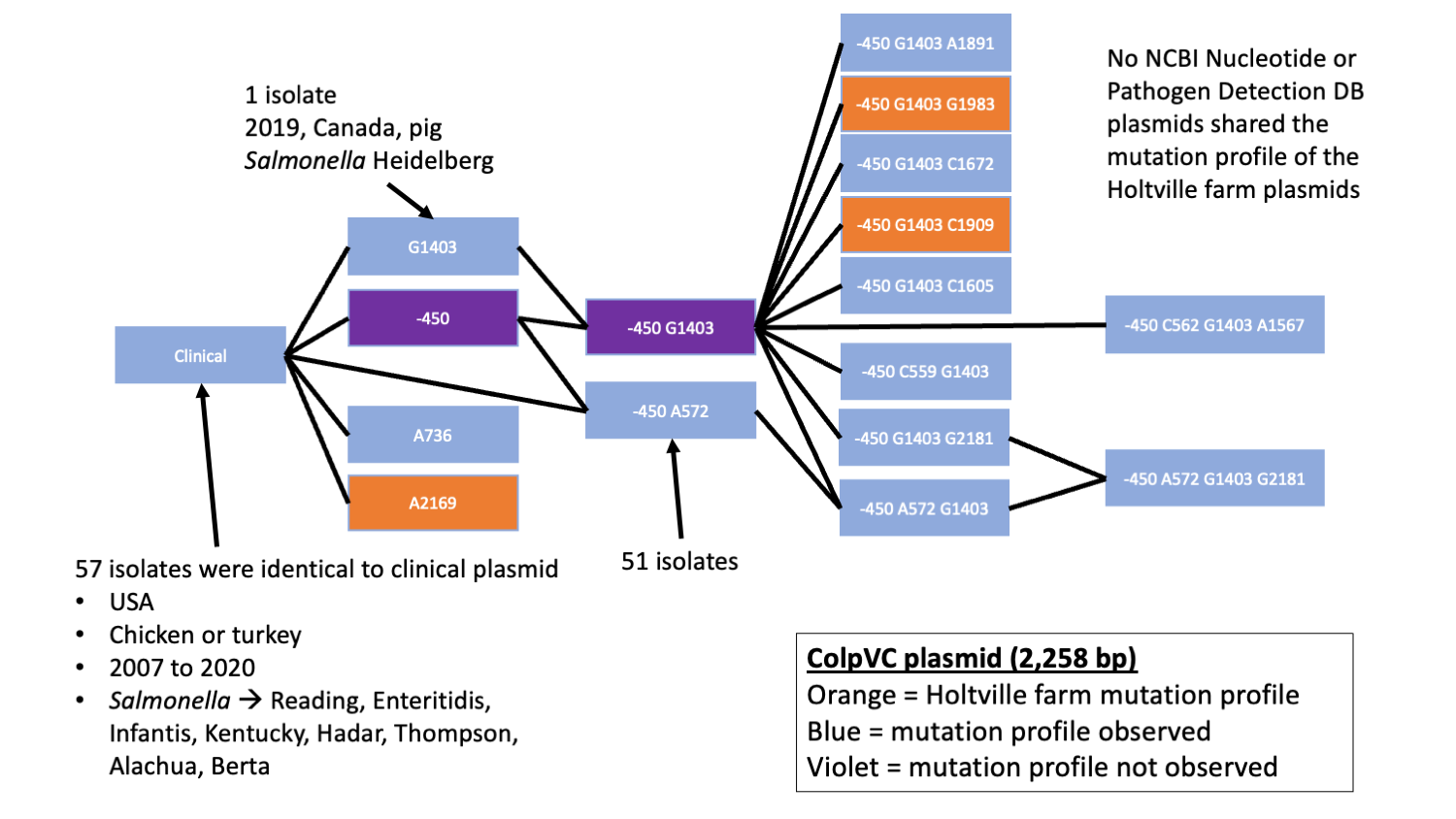


Supplementary Figure 3: ColpVC plasmid mutation graph using the clinical plasmid as the reference. For example, G1403 means a plasmid was identical to the clinical plasmid except a mutation to G at loci 1403 (a “-“ mutation indicates a deletion). The graph was built using the multiple sequence alignment of the plasmids (2,258 bp) which had 2,237 invariant sites. This graph shows mutational differences and does not necessarily represent phylogenetic relationships.


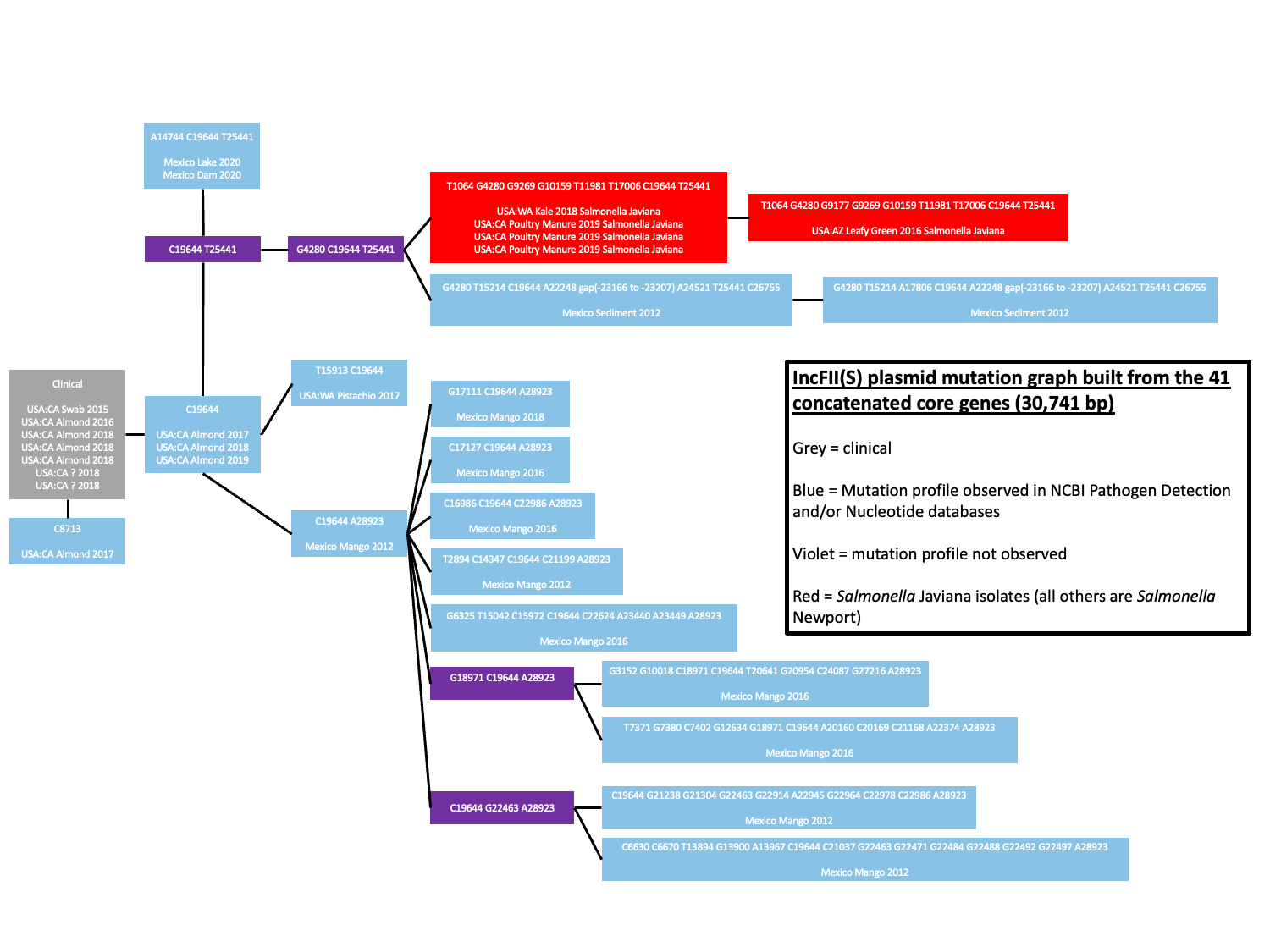
Supplementary Figure 4: The IncFII(S) plasmid mutation graph using the clinical plasmid as the reference. The mutation graph was built from the multiple sequence alignment of the 41 concatenated core genes (30,741 bp) which had 29,882 invariant sites. For example, C8713 indicates a plasmid was identical to the clinical plasmid except for a mutation to C at loci 8713 (a “-“ mutation indicates a deletion). This graph shows mutational differences and does not necessarily represent phylogenetic relationships.

Supplementary Table 1: Summary of the plasmid types, with highly similar instances, observed in both the clinical and farm isolates that were used for statistical analysis. The uncharacterized plasmid was not used because all instances could not be identified in the clinical isolates. Two of the IncI1-I(Gamma) plasmids were not considered highly similar for this analysis because they did not fall into the same subclade as the farm instances.

| Plasmid type of highly similar clinical and farm plasmids | Total number of clinical plasmids | Number of highly similar clinical and farm plasmids (clinical, farm) | Minimum percent identity between full length alignments of highly similar clinical and farm plasmids |
| --- | --- | --- | --- |
| ColpVC | 12 | 5 (1, 4) | 99.89% |
| Col440I | 3 | 7 (1, 6) | 99.87% |
| IncI1-I(Gamma) | 27 | 10 (8, 2) | 98.55% |
| Uncharacterized | 373 | 7 (2, 5) | 95.41% |
